# Factors Influencing the Willingness of Layer Specialized Households to Participate in Cooperative Avian Influenza Prevention and Control: Evidence from China

**DOI:** 10.3390/vetsci12121194

**Published:** 2025-12-12

**Authors:** Donghao Guo, Hua Pu

**Affiliations:** Institute of Animal Sciences, Chinese Academy of Agricultural Sciences, Beijing 100193, China; guodonghao@caas.cn

**Keywords:** avian influenza, cooperative prevention and control, layer specialized households, ordered probit model, regional differences

## Abstract

Background: Highly pathogenic avian influenza (HPAI) remains a significant threat to poultry production in China. Layer specialized households (LSHs)—characterized by medium-scale operations (1000–15,000 birds)—are particularly vulnerable due to frequently suboptimal biosecurity measures. Cooperative prevention and control (CPC) strategies, including unified disinfection and joint monitoring, present a viable method for reducing HPAI risks. However, participation among LSHs remains low. Objectives: This study seeks to identify the key determinants influencing LSHs’ willingness to participate in CPC measures against HPAI, and further compare these driving factors across villages with versus without a documented history of HPAI outbreaks. Methods: A survey of 130 LSHs was conducted in two Chinese villages: Village A (with HPAI history) and Village B (without HPAI history). Data on socio-economic characteristics, production practices, and attitudes were collected via structured questionnaires. An Ordered Probit model was employed to analyze determinants of willingness to participate, measured on a 5-point ordinal scale. Results: Full-sample regression analysis demonstrated that older age, higher educational attainment, risk-tolerant attitudes, larger household scale, and higher annual household income exerted a significantly positive impact on the willingness to participate in the program. Surprisingly, greater knowledge of avian influenza exerted a significant negative effect. Regional subgroup analyses identified distinct drivers: in Village A (with HPAI history), prior experience of bird deaths positively influenced willingness, whereas longer breeding experience had a negative effect. In Village B (no HPAI history), frequent social interaction and greater breeding experience emerged as positive determinants. Conclusions: The findings underscore the critical role of regional context and specific household characteristics in shaping LSHs’ willingness to cooperate. Policy interventions to promote CPC should be regionally tailored: in previously affected areas, leveraging past outbreak experiences and ensuring equitable compensation is essential; in unaffected areas, fostering social networks and demonstrating CPC benefits through peer influence are more effective.

## Simple Summary

Highly Pathogenic Avian Influenza (HPAI) poses a severe threat to China’s layer industry, particularly to Layer Specialized Households (LSHs)—medium-scale farms (1000–15,000 birds) that lack robust biosecurity and are critical to national egg supply. Cooperative prevention and control (CPC) strategies (e.g., unified disinfection, joint monitoring) offer a promising solution to mitigate HPAI risks, yet LSH participation remains low. This study surveyed 130 LSHs across two Chinese villages—one with a documented HPAI history and one without—to identify factors influencing their willingness to engage in CPC. Using an Ordered Probit model, we found that age, educational attainment, risk tolerance, household size, and annual income positively predicted participation willingness in the full sample. Notably, greater avian influenza knowledge unexpectedly exerted a negative effect, potentially due to “free-rider” concerns or overconfidence in independent prevention. Regional differences were pronounced: in the village with HPAI outbreaks, prior bird death experience boosted willingness, while longer breeding experience reduced it; in the unaffected village, frequent social interaction and breeding experience emerged as key drivers. These findings highlight the need for context-specific policies—leveraging outbreak experiences and compensation in affected areas, and fostering social networks in unaffected regions—to enhance cooperative HPAI prevention among LSHs, thereby safeguarding food security, rural livelihoods, and public health.

## 1. Introduction

China dominates global egg production, with national poultry egg output reaching 35.88 million metric tons in 2024 [1]. As a crucial source of animal protein, the layer industry—particularly the segment consisting of Layer Specialized Households (LSHs)—faces persistent threats from Highly Pathogenic Avian Influenza (HPAI). Defined as farms housing 1000–15,000 layers, LSHs occupy an intermediate niche between smallholders and large-scale enterprises. However, they frequently lack robust biosecurity infrastructure, making them high-risk hotspots for HPAI transmission and potential zoonotic spillover [2,3].

Highly Pathogenic Avian Influenza (HPAI), particularly subtypes such as H5N1, poses a persistent and multi-faceted threat to the global poultry industry, public health security, and socio-economic stability [4]. In China, targeted prevention and control measures for the layer hen industry are of paramount importance, with their significance manifested in the following three dimensions:

Safeguarding Food Security and Protein Supply: The layer industry is a critical component of China’s animal husbandry sector. Eggs serve as an affordable, high-quality primary source of animal protein in the daily diet of Chinese residents [5]. HPAI outbreaks can lead to large-scale mortality and culling of layer hens, directly triggering egg supply shortages and sharp price fluctuations—consequences that severely undermine national food security and the stability of people’s livelihoods [6,7].

Preventing Significant Economic Losses: HPAI outbreaks inflict devastating economic impacts on farmers, encompassing both direct losses (e.g., mortality of poultry, compensation for culling) and indirect losses (e.g., trade disruptions, temporary market closures) [8]. For smallholder farmers whose livelihoods depend entirely on layer hen breeding, such losses may push them into poverty or debt [9].

Reducing Zoonotic and Pandemic Risks: HPAI viruses exhibit inherent potential for cross-species transmission [10]. Layer hen farms, as hotbeds for viral replication and transmission, increase the likelihood of viral mutation and recombination in environments with close human–animal interaction [11]. Previous studies have identified live poultry markets and high-density layer hen farms as critical interfaces facilitating the cross-species spillover of avian influenza viruses to humans [12]. Although the H5N1 subtype has not yet acquired efficient human-to-human transmission capacity, each human infection case provides an opportunity for the virus to adapt to the human host, thereby posing a potential risk of global pandemic [13]. Therefore, source-oriented control of HPAI in layer hens serves as the first line of defense for protecting public health and safety.

Vaccination constitutes another indispensable pillar in China’s HPAI prevention and control system, particularly for the layer hen industry. As the first country to implement nationwide compulsory vaccination against H5-subtype avian influenza, China has maintained high immunization coverage in commercial poultry flocks, which has played a decisive role in reducing viral prevalence, limiting inter-farm transmission, and averting large-scale outbreaks [14]. Evidence shows that when properly administered, vaccination significantly reduces viral shedding, lowers morbidity and mortality, and mitigates zoonotic spillover risks [15]. However, vaccine effectiveness depends heavily on antigenic matching, timely booster administration, and standardized inoculation practices. In reality, LSHs often face challenges such as irregular vaccination schedules, insufficient cold-chain management, and inadequate technical capacity [16]. These deficiencies may create immunity gaps that allow viruses to circulate silently or evolve into vaccine-escape variants [17]. Strengthening immunization management among small- and medium-scale farmers is therefore critical for sustaining flock immunity and ensuring the long-term effectiveness of China’s vaccination strategy.

Despite the presence of modern large-scale breeding enterprises in China, layer hen farmers—primarily small and medium-scale breeders—remain the backbone of the industry and have emerged as the most complex and vulnerable link in HPAI prevention and control [18]. Literature indicates that China’s poultry production system adopts a dual-structure characterized by “leading large-scale enterprises and dominant small-to-medium-scale farmers” [19]. A notable feature of this system is the widespread existence of “specialized layer-raising villages,” where dozens or even hundreds of small-to-medium-scale layer hen farmers are geographically concentrated [20]. While this model offers advantages such as operational flexibility and low production costs, it also creates a unique epidemiological environment conducive to viral spread [18,21].

Compared with large-scale enterprises, small-to-medium-scale farmers face multiple challenges in implementing biosecurity measures, primarily reflected in the following aspects:

Inadequate infrastructure investment: Most smallholders lack sufficient funds to establish robust physical barriers (e.g., anti-bird nets), standardized disinfection facilities, and dedicated isolation zones for sick poultry [18].

Knowledge and practice gaps: Studies have shown that small-scale farmers generally exhibit low awareness of the importance of biosecurity measures and limited capacity to implement them [22]. Common issues include improper disposal of dead poultry, lax visitor access management, and mixed breeding of poultry from different batches—all of which increase viral transmission risks [23].

Dense breeding layout: In specialized layer-raising villages, farms are highly concentrated, with chicken coops often separated only by a single wall [24]. Research has confirmed that this high-density breeding pattern significantly facilitates mechanical transmission of viruses between farms via aerosols, contaminated equipment, personnel movement, or vehicles, forming a “high-risk epidemiological landscape” [25].

Due to the aforementioned challenges, individual prevention and control measures implemented by single farmers have limited effectiveness in densely populated breeding villages [26]. Viruses can easily infiltrate through any farm with weak biosecurity and rapidly spread to the entire village [27]. Thus, cooperative epidemic prevention is both crucial and urgent.

The effectiveness of HPAI prevention and control depends on addressing the “weakest link” in the system [28]. Only when all farmers in a community or village adopt coordinated and consistent biosecurity measures can the community transmission chain of the virus be effectively interrupted [29]. Such collective actions include establishing unified disinfection channels, sharing real-time epidemic information, coordinating the timing of breeding stock introduction and slaughter, and conducting centralized harmless disposal of sick and dead poultry [30,31].

This study addresses these gaps by: (1) focusing specifically on LSHs as a vulnerable yet understudied group; (2) explicitly comparing factors influencing CPC willingness between villages with and without HPAI outbreak history; and (3) employing an Ordered Probit model to analyze ordinal willingness data. The findings aim to furnish evidence for developing targeted policies to improve HPAI prevention and control efficiency within China’s layer industry.

### Literature Review and Research Objectives

Existing studies on Highly Pathogenic Avian Influenza (HPAI) prevention, control, and farmer cooperation can be grouped into three main strands, yet important gaps remain concerning specialized poultry households and regional heterogeneity.

The first strand employs empirical and modeling approaches to examine how production practices, contact networks, and biosecurity measures shape HPAI transmission. Paul et al. [32] show that disinfection during trading significantly reduces H5N1 infection in Thai backyard farms, while economic-epidemiological models reveal that control measures—particularly culling and compensation—may generate unintended behavioral responses [33], including rapid restocking or “overstocking,” thereby complicating long-term epidemic control. Outbreak-induced volatility in poultry prices [34] and guidance from FAO [6] and WOAH [35] on early detection, transport-link biosecurity, and cross-sector collaboration further underscore the need to balance epidemiological goals with farmers’ economic incentives.

The second strand focuses on social capital and collective action mechanisms. Research grounded in collective action theory indicates that social norms and peer effects often predict cooperative behavior more reliably than trust alone [36]. Evidence from resource management and agricultural incentive programs shows that network embeddedness, perceived norms, and organizational involvement significantly influence participation [37]. Surveys of small-scale farmers reveal that distrust in government, concerns about compulsory culling, and differing information channels markedly affect compliance and cooperative willingness [28,33], highlighting the importance of communication and trust-building alongside technical incentives.

The third strand—centered on China and other developing countries—uses survey data and econometric models [38] to identify micro-level determinants of biosecurity adoption and cooperative participation. Key findings include positive effects of economic and human capital, the role of training and professional services, and pronounced regional heterogeneity, as outbreak exposure reshapes risk perception and institutional trust. Yet, despite evidence from McLaughlin et al. [39] showing that outbreak experience and regional prevalence significantly affect farmers’ cooperative willingness, regional outbreak history is seldom incorporated into analytical frameworks in the Chinese context.

Addressing these gaps, this study focuses on two underexplored areas: (1) the neglect of heterogeneity among layer farms—particularly Layer Specialized Households (LSHs)—in existing research; and (2) the limited integration of regional differences in HPAI outbreak history when examining cooperative willingness. Accordingly, the study aims to identify key determinants of LSHs’ willingness to participate in cooperative HPAI prevention and control, compare these determinants across outbreak and non-outbreak villages, and propose evidence-based policy recommendations to support effective cooperative models.

## 2. Materials and Methods

### 2.1. Study Area and Sampling

Two villages in China were purposively selected based on their HPAI history:

Village A: Located in a traditional layer-farming region, experienced two confirmed HPAI outbreaks (2008 and 2012). The first outbreak occurred in May 2008, was confirmed as H5N1 avian influenza, and led to the culling of over 30,000 birds to prevent further spread; the second outbreak occurred in April 2012, was confirmed as H5N1 avian influenza, resulting in over 23,800 infected laying hens and the culling of approximately 95,000 birds as part of emergency containment measures. A total of 72 households were surveyed in this village, yielding 70 valid responses (response rate: 97.2%).

Village B: An emerging layer-farming region with no recorded HPAI history. Eighty households were surveyed, yielding 60 valid samples (response rate: 75.0%). To verify that Village B had no history of HPAI outbreaks, this study adopted a multi-source validation approach. First, we confirmed this information through interviews with official staff at the township veterinary station, who are responsible for routine surveillance, vaccination supervision, and outbreak investigations. They verified that Village B has never been designated as an HPAI outbreak area or high-risk area. Second, to further enhance the reliability of this assessment, we conducted key informant interviews with village-level biosecurity personnel and long-term layer farmers. None of the respondents reported any HPAI-related mortality, culling, or emergency response activities.

Stratified census sampling was employed to cover all specialized households (maintaining 1000–15,000 birds) in both villages, ensuring sample representativeness. Face-to-face interviews were conducted using a structured questionnaire, which was pretested and validated (Cronbach’s α = 0.78).

### 2.2. Model Specification

The dependent variable—“willingness to participate in cooperative prevention and control”—is an ordinal categorical variable (1 = Unwilling; 2 = Slightly Unwilling; 3 = Willing; 4 = Moderately Willing; 5 = Strongly Willing). Given the ordered nature of the response variable, an Ordered Probit model was selected over Ordinary Least Squares regression.

#### 2.2.1. Model Formulation

The model specifies a latent continuous variable $y_{i}^{*}$ representing the unobserved propensity of the (i)−th LSH to participate in CPC:

$$y_{i}^{*}=X_{i}\beta +\varepsilon_{i}$$

where $\left(X_{i}\right)$ is a vector of explanatory variables for household i,β is a vector of parameters to be estimated, and $\varepsilon_{i}$ is a random error term assumed to follow a standard normal distribution.

The observed ordinal response $\left(y_{i}\right)$ is linked to the latent variable via threshold parameters $\left(\mu_{1}<\mu_{2}<\mu_{3}<\mu_{4}\right)$:

$$\begin{array}{ll} & 1\;\;if\;y_{i}^{*}\leq \mu_{1}\\ & 2\;\;if\;\mu_{1}<y_{i}^{*}\leq \mu_{2}\\ y_{i}= & 3\;\;if\;\mu_{2}<y_{i}^{*}\leq \mu_{3}\\ & 4\;\;if\;\mu_{3}<y_{i}^{*}\leq \mu_{4}\\ & 5\;\;if\;y_{i}^{*}>\mu_{4} \end{array}$$

#### 2.2.2. Estimation

Parameters $\left(\beta ,\mu \right)$ were estimated using Maximum Likelihood Estimation (MLE) in Stata 18.0.

### 2.3. Variable Definition and Assignment

Drawing upon existing literature and field observations, 17 explanatory variables were selected and categorized into four groups: individual characteristics, household characteristics, production characteristics, and regional/social characteristics (see Table 1).

Key variables included age, education level, risk attitude, knowledge of AI, household size, annual income, farm size, breeding experience, social interaction frequency, and history of bird deaths.

### 2.4. Data Analysis

Statistical analyses were performed using Stata 18.0. Key diagnostic tests included:

Parallel lines test: Confirmed the proportional odds assumption of the Ordered Probit model (*p* = 0.23 > 0.05), supporting model validity.

Multicollinearity test: All variance inflation factors (VIF) were below 5, indicating no severe multicollinearity.

Goodness of fit: Pseudo *R^2^* = 0.124, which is acceptable for cross-sectional behavioral data.

We confirm that one of the two repeated Section 2.4 contents should be deleted.

## 3. Results

### 3.1. Sample Characteristics

Table 2 summarizes the socioeconomic characteristics of the surveyed LSHs.

The two sampled villages exhibited distinct socioeconomic profiles despite sharing similar demographic baselines. Both were dominated by middle-aged male farmers with junior-high education, yet Village B showed a markedly lower human-capital structure, with 36.2% having only primary schooling compared with 22.9% in Village A.

Clear inter-village differences emerged in age composition and production experience. Village B had a substantially younger farming cohort (37.5% aged 25–39), whereas Village A was characterized by an older and more experienced population—64.3% had more than five years of breeding experience, compared with only 25.0% in Village B. These differences align with the developmental stages of the two regions: Village A as a long-established production base and Village B as an emerging farming cluster.

Production scale and income distributions further underscored the structural divergence. Village A remained dominated by small-scale operations, with 45.7% maintaining only 1000–2000 birds. In contrast, Village B displayed pronounced scale intensification: 41.2% kept 4001–8000 birds and an additional 8.8% operated at 8001–15,000 birds. Income patterns followed the same gradient—45.7% of Village A households earned just 10,000–30,000 RMB annually, whereas 37.5% of Village B households reached 70,000–90,000 RMB and 13.8% surpassed 100,000 RMB.

Family labor structures were largely comparable, though Village B had a higher share of households with three or more laborers (10.0% vs. 2.9%).

Overall, Village A represents a traditional, small-scale, low-income but experience-rich production system, whereas Village B reflects a newer, rapidly expanding, higher-income layer-farming cluster. These sharp structural contrasts provide essential contextual foundations for interpreting heterogeneity in cooperative prevention behavior and CPC willingness across the two communities.

### 3.2. Ordered Probit Model Estimation Results

#### 3.2.1. Full-Sample Results

Positive determinants: Willingness increases significantly with age (β = 0.042, *p* < 0.01), risk-tolerant attitude (β = 0.169, *p* < 0.05), household size (β = 0.167, *p* < 0.1), and annual income (β = 0.064, *p* < 0.05). These patterns suggest that older, better-resourced households—both in labor and financial capacity—exhibit a stronger propensity to engage in coordinated action. Education exerts a marginally positive effect (β = 0.323, *p* < 0.1).

Negative determinant: A notable negative influence arises from AI knowledge (β = −0.187, *p* < 0.05). This counterintuitive effect indicates that better-informed farmers may perceive lower marginal benefits from collective prevention, either due to stronger confidence in self-protection or a heightened awareness of coordination costs.

Non-significant factors: Farm scale, cooperative affiliation, and previous AI outbreaks do not exert statistically identifiable effects in the aggregate model, implying that structural production characteristics alone do not universally drive preventive cooperation.

#### 3.2.2. Village-Specific Results

Substantial spatial heterogeneity emerges once the sample is stratified by epidemiological context. Village A, with documented HPAI history, displays a risk- and experience-driven pattern, whereas Village B, an emerging production cluster with no outbreak history, is shaped primarily by social and economic factors.

Village A (with HPAI history): In the epidemic-experienced setting, farmers’ willingness to participate in cooperative HPAI prevention is jointly shaped by a configuration of risk perception, direct loss experience, and access to professional expertise. Willingness rises significantly with age (β = 0.086, *p* < 0.01), risk-tolerant attitudes (β = 0.231, *p* < 0.05), and household size (β = 0.330, *p* < 0.05), while two context-specific drivers exert particularly strong positive effects: previous bird-mortality experience (β = 0.656, *p* < 0.1), which indicates that direct biological losses heighten the perceived benefits of coordinated action, and veterinary consultation (β = 0.477, *p* < 0.1), underscoring the critical role of professional information in motivating cooperation among risk-aware farmers. At the same time, two experience-based factors operate as negative determinants. Breeding years (β = −0.108, *p* < 0.1) reduce willingness, suggesting that more seasoned farmers tend to rely on established private prevention strategies rather than collective mechanisms, while AI knowledge (β = −0.312, *p* < 0.05) similarly dampens cooperation, reinforcing the interpretation that self-assessed competence may render coordinated arrangements less attractive. Taken together, Village A exhibits a distinct risk–experience–expertise nexus, wherein past exposure strengthens cooperative inclinations through loss memory and professional engagement, yet simultaneously fosters a form of self-reliant confidence that partially substitutes for collective action.

Village B (no HPAI history): In contrast, the emerging production cluster exhibits a markedly different behavioral structure characterized by a social-network and resource-capacity pattern, rather than a risk-driven one. Cooperative willingness increases significantly with social interaction frequency (β = 0.372, *p* < 0.1), annual income (β = 0.074, *p* < 0.05), and breeding years (β = 0.204, *p* < 0.05), indicating that in settings without prior epidemic exposure, participation in collective prevention is primarily rooted in social embeddedness and economic capacity. Consistent with the absence of risk memory, no significant negative determinants are identified for Village B, suggesting that farmers’ decision-making is shaped predominantly by social connectivity and resource endowments rather than by deterrents or conflicting incentives.

The heterogeneous results (Table 3) demonstrate that cooperative preventive behavior is fundamentally context-contingent, shaped by markedly different mechanisms across epidemiological settings. In regions with prior epidemic experience, willingness to participate in cooperative measures is driven by heightened risk perception, direct loss exposure, and the reinforcing role of veterinary professionals, whereas accumulated breeding experience and AI-related knowledge tend to substitute for collective action incentives. By contrast, in emerging and outbreak-free clusters, decision-making is governed primarily by social-network embeddedness and household economic endowments rather than by risk-based motivations. Together, these findings underscore the necessity of regionally differentiated intervention strategies that align cooperative prevention programs with local epidemiological histories, information environments, and community social structures.

## 4. Discussion

### 4.1. Key Findings in Context of Existing Literature

#### 4.1.1. The Paradoxical Effect of Avian Influenza Knowledge

Existing studies generally suggest that disease-related knowledge enhances farmers’ biosecurity practices and their willingness to cooperate [33,37]. However, our results show a significant negative association between avian influenza knowledge and the willingness to participate in CPC. This finding aligns with collective action theory: farmers with higher knowledge levels may be more aware of the potential “free-rider” problem in collective schemes and thus have lower trust in the effectiveness of group-based solutions [38]. Meanwhile, the “overconfidence effect” [28] may lead knowledgeable farmers to overestimate their own capacity for independent disease prevention, reducing their reliance on community cooperation.

This evidence suggests that knowledge dissemination alone does not automatically translate into cooperative behavior. Trust-building and transparent cooperative mechanisms must accompany knowledge programs to effectively enhance farmers’ participation in CPC.

#### 4.1.2. Pronounced Regional Differences

Inter-village differences indicate that outbreak experience is a critical contextual factor shaping cooperative behavior. In Village A—where HPAI outbreaks occurred—mortality losses significantly increased farmers’ willingness to cooperate, consistent with the “experiential learning” mechanism. Direct losses heighten risk perception, making farmers more inclined to participate in collective prevention.

In contrast, in Village B—where no outbreak had occurred—cooperation was driven mainly by social interaction and production experience. This pattern reflects a “social learning” process: in the absence of direct experience, farmers rely on peer information and demonstration effects to assess the credibility of cooperative mechanisms.

Therefore, policy interventions should emphasize compensation and trust reconstruction in outbreak-affected areas, while in non-affected areas, strategies should focus on leveraging social networks and demonstration households to promote cooperation.

### 4.2. Practical Implications for Policy

The prevention and control of HPAI among small- and medium-scale poultry farmers is vital for safeguarding China’s food security, stabilizing rural livelihoods, and reducing zoonotic risks [32]. Existing research emphasizes that smallholders—due to high density and limited biosecurity—function as potential amplification nodes during epidemics [41]. Our findings further demonstrate that risk cognition, experience, and social context significantly shape farmers’ willingness to participate in CPC, implying the need for differentiated and behaviorally informed policy interventions.

Targeted Awareness Campaigns: Targeted awareness campaigns should be tailored to local contexts. In areas without outbreak history, case-based narratives from affected regions may elevate risk perception. In previously affected areas, sharing successful cooperative experiences can help counter resistance among experienced farmers who may overvalue independent risk management.

Cooperative Capacity Building: Cooperative capacity building is essential. Concerns about transparency in cost-sharing, information asymmetry, and benefit distribution have been documented as barriers to collective action [37]. Enhancing cooperative governance—through clear rules, regular disclosure, and participatory decision-making—may improve perceived fairness and credibility.

Financial Incentives: Financial incentives such as subsidies for disinfection, vaccination checks, or antibody testing can help alleviate cost burdens, particularly for lower-income households in emerging poultry regions. Prior studies show that targeted subsidies significantly improve compliance and community participation in animal-health interventions [38].

### 4.3. Limitations and Future Research

This study has several limitations. First, while the sample is representative of the selected villages, its relatively small size (*n* = 130) and geographical focus limit the generalizability of the findings. Future research should expand the sampling frame to include major layer-producing provinces across China. Second, the cross-sectional design precludes causal inference. Longitudinal or panel studies are needed to track evolutionary changes in willingness, particularly following an HPAI outbreak. Future investigations could also integrate experimental methods (e.g., trust games) to quantitatively assess the impact of trust dynamics on cooperative behavior.

## 5. Conclusions

This study identifies the determinants of Layer Specialized Households’ willingness to participate in HPAI cooperative prevention and control using an Ordered Probit modeling framework. The core findings demonstrate that the drivers of cooperation are not universal but are significantly shaped by the local epidemiological context. In regions with a history of HPAI, policy interventions should capitalize on the salience of past losses and ensure robust, transparent compensation mechanisms. In areas without prior outbreaks, building social capital and leveraging peer networks are paramount. Tailored interventions that integrate these context-specific insights with strategic financial support and transparent cooperative governance are essential for strengthening the overall resilience of China’s layer industry against HPAI threats.

## Figures and Tables

**Table 1:** Definition and Assignment of Variables.

Variable Category	Variable Code	Variable Name	Definition and Assignment	References
Dependent Variable	*Y*	Cooperative Willingness	1 = Unwilling; 2 = Slightly Unwilling; 3 = Willing; 4 = Moderately Willing; 5 = Strongly Willing	[9]
Individual Characteristics	*X1*	Gender	1 = Male; 0 = Female	[9]
	*X2*	Age	Continuous variable (actual age, years)	[9]
	*X3*	Education Level	1 = Primary school or below; 2 = Junior high school; 3 = Senior high school or above	[9]
	*X4*	AI Knowledge	Score of 4 questions (1 point for each correct answer): ① AI infects humans? (1 = Yes, 0 = No) ② Distinguish IB vs. ND? (1 = Yes, 0 = No) ③ Distinguish ND vs. AI? (1 = Yes, 0 = No) ④ AI virus in feces? (1 = Yes, 0 = No)	[9]
	*X5*	Risk Attitude	1 = Not daring to try new tech; 2 = Less daring; 3 = Neutral; 4 = Relatively daring; 5 = Fully daring	[9]
	*X6*	Social Interaction	1 = Rarely interact with strangers; 2 = Less frequent; 3 = Neutral; 4 = Frequent; 5 = Very frequent	[9]
Household Characteristics	*X7*	Household Size	Continuous variable (actual number of family members)	[40]
	*X8*	Annual Income	Continuous variable (10,000 RMB)	[40]
	*X9*	Organizational Awareness	Score (0–4): 2 points for Party member/cadre (self + spouse); 0 = Neither is Party member/cadre	[40]
Production Characteristics	*X10*	Breeding Years	Continuous variable (actual years of layer farming)	[40]
	*X11*	Stocking Scale	Continuous variable (actual number of layers, birds)	[40]
	*X12*	Cooperative Membership	1 = Member of layer cooperative; 0 = Non-member	[40]
	*X13*	Previous AI Outbreak	1 = Birds died of AI before culling; 0 = No death	[40]
	*X14*	Neighbor Distance	Continuous variable (distance to adjacent farm, meters)	[40]
	*X15*	Vehicle Disinfection	1 = Disinfect chicken traders’ vehicles every time; 0 = Not every time	[40]
	*X16*	Veterinary Consultation	1 = Consult vet every time; 2 = Consult vet most times; 3 = Rarely consult vet	[40]
Regional Characteristics	*X17*	Regional AI History	1 = Village with AI outbreaks; 0 = Village without AI outbreaks	[41]

**Table 2:** Sample Characteristics (Percentage, %).

Characteristic	Category	Village A	Village B	Characteristic	Category	Village A	Village B
Gender	Male	85.7	90.0	Stocking Scale (birds)	1000–2000	45.7	0.0
	Female	14.3	10.0		2001–4000	47.2	50.0
Age (Years)	25–39	24.3	37.5		4001–8000	7.1	41.2
	40–49	42.9	47.5		8001–15,000	0.0	8.8
	50–59	31.4	11.2	Annual Income (10k RMB)	1–3	45.7	18.6
	60–74	1.4	3.8		4–6	44.3	27.1
Education	Primary or below	22.9	36.2		7–9	10.0	37.5
	Junior high	55.7	53.8		10–19	0.0	13.8
	Senior high or above	21.4	10.0		20–43	0.0	7.5
Breeding Years	1–3	10.0	41.2	Family Labor (person)	≤1	8.6	7.5
	4–5	25.7	33.8		2	88.6	82.5
	>5	64.3	25.0		≥3	2.9	10.0

**Table 3:** Ordered Probit Model Estimation Results.

Variable	Full Sample	Village A	Village B
	Coefficient (*p*-Value)	Coefficient (*p*-Value)	Coefficient (*p*-Value)
Y (Mean)	3.89	3.98	3.80
X1 (Gender)	0.074 (0.813)	−0.619 (0.227)	0.334 (0.542)
X2 (Age)	0.042 *** (0.008)	0.086 *** (0.002)	0.024 (0.361)
X3 (Education)	0.323 * (0.090)	0.269 (0.302)	0.243 (0.517)
X4 (AI Knowledge)	−0.187 * (0.054)	−0.312 ** (0.039)	−0.074 (0.646)
X5 (Risk Attitude)	0.169 ** (0.022)	0.231 ** (0.025)	0.125 (0.349)
X6 (Social Interaction)	0.106 (0.359)	0.129 (0.523)	0.372 * (0.081)
X7 (Household Size)	0.167 * (0.072)	0.330 ** (0.028)	0.194 (0.215)
X8 (Annual Income)	0.064 ** (0.032)	−0.086 (0.504)	0.074 ** (0.046)
X9 (Organizational Awareness)	0.008 (0.974)	−0.026 (0.932)	0.504 (0.515)
X10 (Breeding Years)	−0.019 (0.640)	−0.108 * (0.085)	0.204 * (0.073)
X11 (Stocking Scale)	0.0001 (0.125)	0.0002 (0.264)	0.0001 (0.225)
X12 (Cooperative Membership)	0.061 (0.818)	0.126 (0.744)	−0.819 (0.121)
X13 (Previous AI Outbreak)	0.391 (0.202)	0.656 * (0.067)	—
X14 (Neighbor Distance)	0.0003 (0.391)	0.0014 (0.132)	0.0004 (0.388)
X15 (Vehicle Disinfection)	−0.201 (0.401)	−0.282 (0.406)	−0.471 (0.307)
X16 (Veterinary Consultation)	0.141 (0.430)	0.477 * (0.054)	−0.638 (0.137)
X17 (Regional AI History)	0.441 (0.239)	—	—
Observations	130	70	60
Pseudo R^2^	0.124	0.198	0.183
Prob > chi^2^	0.002	0.012	0.026

Note: *** *p* < 0.01, ** *p* < 0.05, * *p* < 0.1; “—” indicates no applicable data.

## Data Availability

No new data were created or analyzed in this study. Data sharing is not applicable to this article.
